# Patient-controlled intravenous analgesia with opioids after thoracoscopic lung surgery: a randomized clinical trial

**DOI:** 10.1186/s12871-022-01785-4

**Published:** 2022-08-08

**Authors:** Hong Yu, Wei Tian, Zhao Xu, Rongjuan Jiang, Liang Jin, Wenjie Mao, Ying Chen, Hai Yu

**Affiliations:** 1 Department of Anesthesiology, West China Hospital, Sichuan University. No, 37 Guoxue Alley, Chengdu, 610041 China; 2 Department of Anesthesiology, The First People’s Hospital of Neijiang, Neijiang, 641000 China; 3 Department of Anesthesiology, Chengdu Second People’s Hospital, Chengdu, 610017 China; 4 Department of Anesthesiology, The People’s Hospital of Leshan, Leshan, 614099 China; 5 Department of Anesthesiology, The People’s Hospital of Jianyang, Chengdu, 641499 China; 6 Department of Anesthesiology, Liangshan Hospital of Integrated Traditional and Western Medicine, Xichang, 615099 China

**Keywords:** Thoracoscopic surgery, Postoperative acute pain, Postoperative nausea and vomiting, Opioid

## Abstract

**Background:**

Opioids remain the mainstream therapy for post-surgical pain. The choice of opioids administered by patient-controlled intravenous analgesia (PCIA) for thoracoscopic lung surgery is unclear. This study compared 3 opioid analgesics for achieving satisfactory analgesia with minimal emesis (SAME).

**Methods:**

This randomized clinical trial enrolled patients scheduled for thoracoscopic lung surgery randomized to receive 1 of 3 opioids for PCIA: oxycodone (group O), hydromorphone (group H), and sufentanil (group S). The primary outcome was the proportion of subjects achieving SAME, i.e., no-to-mild pain (pain score < 4/10) with minimal nausea/vomiting (PONV score < 2/4) when coughing during the pulmonary rehabilitation exercise in the first 3 postoperative days.

**Results:**

Of 555 enrolled patients, 184 patients in group O, 186 in group H and 184 in group S were included in the final analysis. The primary outcome of SAME was significantly different among group O, H and S (41.3% vs 40.3% vs 29.9%, *P* = 0.043), but no difference was observed between pairwise group comparisons. Patients in groups O and H had lower pain scores when coughing on the second day after surgery than those in group S, both with mean differences of 1 (3(3,4) and 3(3,4) vs 4(3,4), *P* = 0.009 and 0.039, respectively). The PONV scores were comparable between three groups (*P* > 0.05). There were no differences in other opioid-related side effects, patient satisfaction score, and QoR-15 score among three groups.

**Conclusions:**

Given clinically relevant benefits detected, PCIA with oxycodone or hydromorphone is superior to sufentanil for achieving SAME as a supplement to multimodal analgesia in patients undergoing thoracoscopic lung surgery.

**Trial registration:**

This study was registered at (ChiCTR2100045614, 19/04/2021).

**Supplementary Information:**

The online version contains supplementary material available at 10.1186/s12871-022-01785-4.

## Introduction

Although minimally invasive techniques are increasingly popular, postoperative pain is one of the most common complaints from patients undergoing video-assisted thoracoscopic surgery (VATS). Of note, patients can still experience moderate-severe pain after VATS [1, 2]. Pain relief after VATS continues to be a challenging issue for anesthesiologists caring for these patients. Satisfactory postoperative pain management is crucial to assuring good patient experience, optimizing postoperative outcomes, enhancing functional recovery after surgery, and potentially decreasing the risk of developing chronic pain [3].

Current approaches for postoperative pain management, are increasingly centered on non-opioid approaches after VATS. For instance, epidurals have classically been recognized as the gold standard for pain management in thoracic surgery [4] and more recently, paravertebral blocks [5, 6]. Although the concept of opioid sparing, or multimodal analgesia has been recommended by guidelines and widely practiced [7–9], the use of patient-controlled intravenous analgesia (PCIA) with opioid is generally the method of choice for pharmacologic pain control [10]. Moreover, opioid analgesics are the historical mainstay for postoperative cardiothoracic surgery pain relief and remain an essential and reliable analgesic for treating moderate to severe pain [11, 12]. However, opioid for postoperative analgesia is a matter of dispute in contemporary practice. Opioid administration is of concern and accompanied with many adverse effects, including postoperative nausea and vomiting (PONV), constipation, urinary retention, respiratory depression and delirium [13–16]. Therefore, it is more important to control postsurgical pain while minimizing opioid-related morbidity [17].

Oxycodone, hydromorphone and sufentanil are potent opioid analgesics used to treat postoperative pain [18, 19]. However, no standardized and optimal opioid treatment for postoperative pain after VATS has been established so far. Consequently, there is a need to determine the most appropriate systemic opioid analgesia to control pain after VATS.

We designed a randomized controlled trial (RCT) to compare 3 opioids PCIA for achieving satisfactory analgesia with minimal emesis (SAME) in patients undergoing thoracoscopic lung surgery.

## Methods

### Ethics and registration

This three-arm RCT was conducted in accordance with the Declaration of Helsinki, and approved by the Ethics Committee of West China Hospital of Sichuan University (Ethical number: 2020[1327]). The protocol was registered in the Chinese Clinical Trials Registry (ChiCTR2100045614, 19/04/2021). All participants provided written informed consent before enrollment. The study protocol followed the CONSORT guidelines.

### Participants

We recruited participants scheduled VATS resection of lung nodules at West China Hospital of Sichuan University, from April 2021 to November 2021. Patients were eligible for participation if they met the following criteria: age 18 years or older; American Society of Anesthesiologists statuses I-III; elective thoracoscopic lung surgery. Exclusion criteria were as follows: refusal to participate in the study, known allergies to study drugs or sulfonamides, renal or liver impairment, sleep apnea syndrome, chronic obstructive airway disease, bronchial asthma, ischemic heart disease, pulmonary heart disease, active gastrointestinal ulcer or bleeding or inflammatory bowel disease, pregnancy or breast-feeding, refuse to use PCIA.

### Randomization and blinding

Eligible patients were randomly assigned to one of three experimental groups (oxycodone, hydromorphone and sufentanil) in a 1:1:1 ratio by computer-generated random permuted blocks of size 6. The blocks of random numbers were generated and put in opaque envelopes, each with a screen number on the front of the envelope. Participants, surgeons, and evaluators assessing outcomes were blinded throughout the study. The investigator opened the envelopes before the end of the surgery and prepared the PCIA pump accordingly. The analgesic pump was put into an opaque portable bag and connected at the end of the surgery. The nurses in the post-anesthesia care unit (PACU) and ward who was responsible for the PCIA knew the grouping.

### Anesthesia and intraoperative care

All participants were monitored with electrocardiogram, pulse oximetry, bispectral index electrodes and noninvasive blood pressure. Anesthesia management protocol was based on guidelines for enhanced recovery after lung surgery [8]. General anesthesia was induced with propofol 1.5 ~ 2.0 mg/kg, sufentanil 0.3–0.5ug/kg, cisatracurium 0.2 mg/kg or rocuronium 0.6 mg/kg. After intubation with a double-lumen tube, lung protective ventilation strategies were adopted as described before [20]. Nonsteroidal anti-inflammatory drug (NSAID) (unless contraindicated) was given intravenously before skin incision. During the operation, anesthesia was achieved with propofol or sevoflurane or desflurane to maintain bispectral index level at 40–60. Intraoperative analgesia was provided with remifentanil and sufentanil. Muscle relaxation monitoring was conducted to guide the use of muscle relaxant and the reversal of neuromuscular blockade. A maintenance crystalloid was administrated throughout the procedure at 4–6 ml/kg/h. Prior to dermal closure, multiple-level, single-injection, unilateral intercostal nerve blocks of T3 to T8 with 20 ml of 0.5% ropivacaine were performed by the surgical team under direct thoracoscopic visualization. At the end of surgery, 5 mg tropisetron was injected intravenously.

### Interventions and postoperative management

PCIA was applied to all patients for postoperative pain relief. Patients were randomly assigned to receiving PCIA containing oxycodone 0.5 mg/ml (group O), hydromorphone 0.05 mg/ml (group H) or sufentanil 0.5 μg/ml (group S) combined with tropisetron 5 mg in 100 ml normal saline. The PCIA was set to deliver 4 ml boluses, a 10-min lockout window, no basal infusion dose. All patients in the study were informed beforehand of the PCIA method in the pre-anesthetic appointment. Patients were instructed to press the PCIA in case of emerging pain. PCIA was maintained until patient was discharged from hospital, liquid used up with no request for further PCIA, or withdrawal due to adverse events. Parecoxib i.v. 40 mg was administered every 8 h in the first 3 days after surgery (flurbiprofen 50 mg if contraindicated). If there were numerical rating scale (NRS) pain scores > 3 and no pain relief by pressing the PCIA, the patient was administered i.v. dezocine 5 mg as rescue analgesics. PONV score ≥ 2 were treated with i.v. metoclopramide 10 mg.

### Outcomes

The primary outcome was defined as the proportion of subjects achieving SAME, i.e., no-to-mild pain (pain score < 4) with minimal nausea/vomiting (PONV score < 2) when coughing during the pulmonary rehabilitation exercise in the first 3 postoperative days. Pain score was assessed on an 11-point NRS (0 = no pain, 0 < NRS < 4 (mild pain), 4 ≤ NRS < 7 (moderate pain), NRS ≥ 7 (severe pain), 10 = worst pain imaginable). The PONV score was defined as follows: 0 = no nausea, 1 = mild nausea (no treatment needed), 2 = moderate nausea or retching (may need treatment), 3 = frequent vomiting (controlled with anti-emetics), and 4 = severe vomiting (uncontrolled with anti-emetics). Key secondary outcomes included pain scores at rest and when coughing, and PONV scores within 3 days after surgery. Other secondary outcomes included the proportion of SAME at rest in the first 3 postoperative days, the total dose of opioids in morphine equivalents, the dose of opioids of PCIA in morphine equivalents, quality of recovery-15 (QoR-15) score, other opioid-related adverse events, and expectation pain score fulfilled on POD1-3, patient satisfaction score on pain control and the length of stay (LOS) in hospital.

At the end of surgery, the patients were transferred to PACU. Pain scores and PONV scores were assessed after tracheal tube removal at PACU by a trained nurse. After the patients returned to the ward, the pain score and PONV score were immediately assessed by a trained nurse, and then assessed every 4 h. Within the first 3 days after surgery, patients were followed up by a trained investigator between 17:00 to 19:00 every day. The investigator recorded the patient’s average pain score and PONV score within the past 24 h according to the nursing records. Patient satisfaction score (0 = dissatisfied and 100 = very satisfied) on pain control, QoR-15 score (ranging from 0 to 150), other opioid-related adverse events, and expectation NRS fulfilled by asking the patient whether the pain was acceptable or not were also recorded.

### Sample size calculation

The sample size estimation was based on previous research data in our center, in which.

the proportion of SAME was 33% in patients undergoing thoracoscopic lung surgery with PCIA (sufentanil 3 μg/kg + tropisetron 15 mg + dexmedetomidine 200 μg in 200 ml normal saline, infusion rate of 2 ml/h) [2]. We hypothesized that 50% of patients achieving SAME would be clinically significant when using the trial interventions. The sample size of 555 was calculated by using PASS15.0, with a 2-tailed type I error rate of 0.0167, a power of 80%, and an anticipated 5% exclusion rate.

### Statistical analysis

IBM SPSS Statistics software (version 26.0) was used for statistical analysis. The analysis was conducted by intent-to-treat. Per-protocol analysis of the primary outcome was also performed. Normality of the data was evaluated using the Kolmogorov–Smirnov test. Normal continuous baseline variables are presented as the mean (standard deviation [SD]), and non-normal continuous variables are presented as median and quartiles, categorical variables are presented as numbers and percentages. For global group comparisons of variables, continuous data were analyzed using one-way analysis of variance or the Kruskal–Wallis test as appropriate, and the R*C Chi-square test was used for comparison of proportions. Specifically, generalized estimating equations with robust standard error estimates were used to account for repeated measures of pain scores and QoR-15 scores. For primary and secondary outcomes, if R*C Chi-square test or the Kruskal–Wallis test results were significant, pairwise group comparisons were performed by Chi-square test or Mann–Whitney U tests, and Bonferroni correction was applied. The level of statistical significance was set at *P* < 0.05.

## Results

**Fig. 1:**
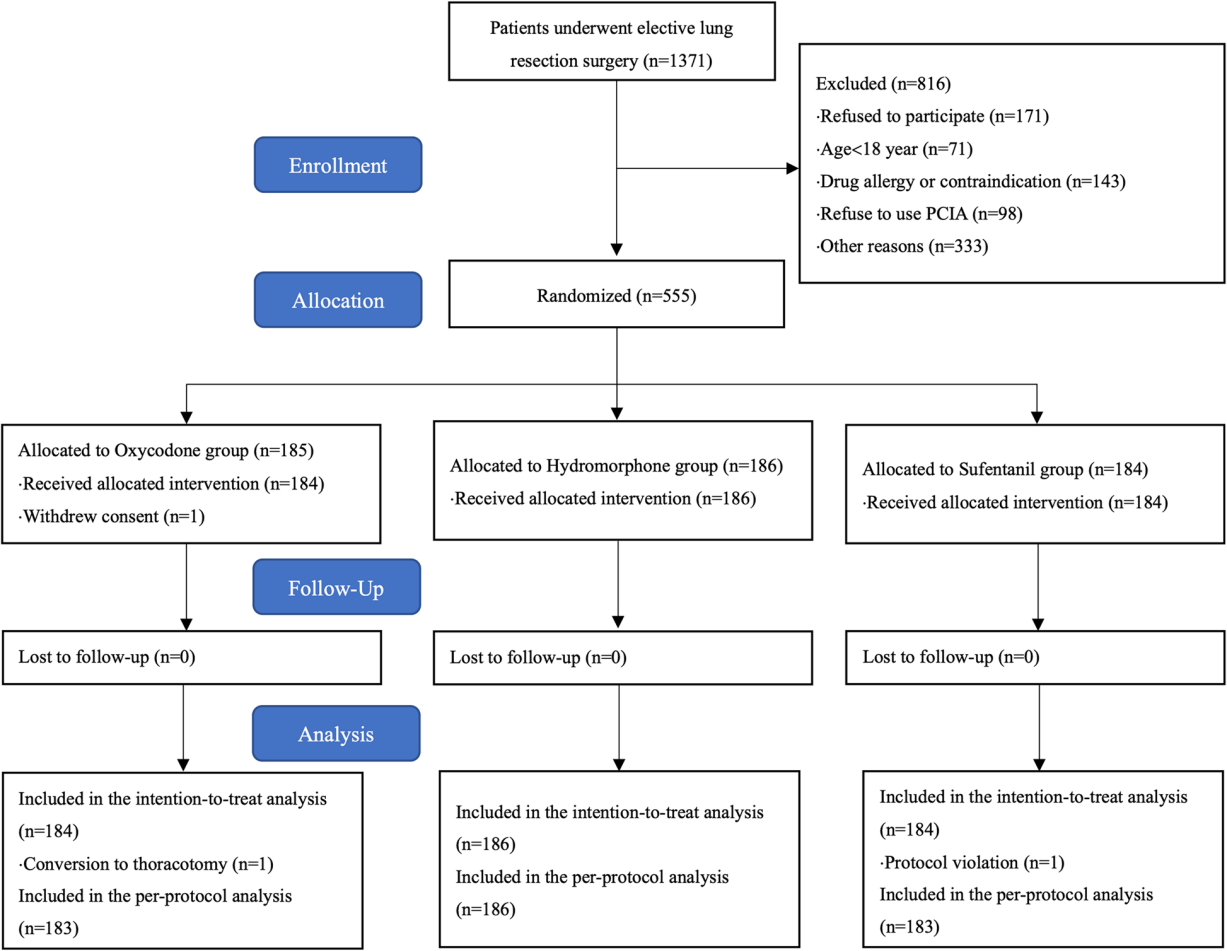
Consort flowchart. PCIA, patient-controlled intravenous analgesia

Of 1371 potentially eligible patients, 555 patients were randomized into the study (Fig. 1). Because one patient in group O withdrew consent after randomization, a total of 554 patients were analyzed with the intent-to-treat principle. One patient was excluded from per-protocol analysis because of conversion to thoracotomy and one protocol violation (not receiving the postoperative analgesia as assigned). The baseline characteristics, and relevant intraoperative and PACU variables are shown in Tables 1 and 2.

**Table 1:** Baseline characteristics of study subjects

Characteristics	Oxycodone group (*n* = 184)	Hydromorphone group (*n* = 186)	Sufentanil group (*n* = 184)	*P* value
Men, no. (%)	74(40.2)	66(35.5)	58(31.5)	0.219
Age, yr, mean (SD)	53.1(10.6)	53.5(12.5)	51.6(12.6)	0.282
BMI, kg/m^2^, mean (SD)	22.93(2.89)	22.94(2.79)	22.71(2.87)	0.677
ASA physical status, no. (%)				0.379
I	1(0.5)	1(0.5)	2(1.1)	
II	171(92.9)	163(87.6)	163(88.6)	
III	12(6.5)	22(11.8)	19(10.3)	
IV-V	0	0	0	
Smoking status, no. (%)				0.295
Never	145(78.8)	157(84.4)	155(84.2)	
Former	36(19.6)	29(15.6)	27(14.7)	
Current	3(1.6)	0(0.0)	2(1.1)	
Apfel risk factors for PONV^a^, no. (%)				0.218
1	38(20.7)	24(12.9)	24(13.0)	
2	38(20.7)	41(22.0)	37(20.1)	
3	103(56.0)	111(59.7)	118(64.1)	
4	5(2.7)	10(5.4)	5(2.7)	
History of chronic pain, no. (%)	9(4.9)	13(7.0)	20(10.9)	0.089
Hypertension, no. (%)	30(16.3)	27(14.5)	31(16.8)	0.814
Diabetes, no. (%)	14(7.6)	14(7.5)	8(4.3)	0.350
Chronic bronchitis or emphysema, no. (%)	15(8.2)	25(13.4)	21(11.4)	0.261
Expected maximal pain on cough^b^, median (IQR)	4(4,5)	4(4,5)	4(4,5)	0.831
Expected maximal pain at rest^b^, median (IQR)	2(2,2)	2(2,2)	2(2,2)	0.784
Expected average pain^b^, median (IQR)	3(3,3)	3(3,3)	3(3,3)	0.778
QoR-15 score, median (IQR)	142(139,144)	142(139,144)	143(139,144)	0.539

Data are presented as the mean (SD), median (IQR), or number (%)

Abbreviations: *ASA* American Society of Anesthesiologists, *BMI* body mass index, *IQR* interquartile range, *PONV* postoperative nausea and vomiting, *QoR-15* Quality of Recovery-15 questionnaire, *SD* standard deviation

^a^Apfel risk factors for PONV: female sex, previous history of postoperative nausea and vomiting or motion sickness, being a nonsmoker, and expected use of post-operative opioids for analgesia

^b^Pain rated on a 0–10 numerical rating scale

**Table 2:** Surgery and anesthesia data; pain score, rescue analgesics and PONV in the PACU

Characteristics	Oxycodone group (*n* = 184)	Hydromorphone group (*n* = 186)	Sufentanil group (*n* = 184)	*P* value
Type of surgery, no. (%)				0.693
Lobectomy	85(46.2)	83(44.6)	75(40.8)	
Segmentectomy	47(25.5)	55(29.6)	59(32.1)	
Wedge resection	52(28.3)	48(25.8)	50(27.2)	
Single-port thoracoscopy, no. (%)	59(32.1)	53(28.5)	60(32.6)	0.649
Malignant lesion, no. (%)	131(71.2)	144(77.4)	144(78.3)	0.226
Duration of surgery, min, median (IQR)	91(64,122)	88(70,114)	90(70,117)	0.878
Anesthesia maintenance, no. (%)				0.554
Volatile anesthetics	152(82.6)	152(81.7)	150(81.5)	
Propofol	13(7.1)	21(11.3)	17(9.2)	
Volatile anesthetics combined with propofol	19(10.3)	13(7.0)	17(9.2)	
Dose of sufentanil, μg/kg, median (IQR)	0.5(0.4,0.6)	0.5(0.4,0.6)	0.5(0.5,0.6)	0.029
Dose of remifentanil, μg/kg/min, median (IQR)	0.1(0.1,0.2)	0.1(0.1,0.2)	0.1(0.1,0.2)	0.457
NSAIDs, no. (%)	165(90.7)	166(89.7)	174(95.1)	0.136
Antiemetic, no. (%)				
Tropisetron	173(94.0)	169(90.9)	167(91.3)	0.474
Methylprednisolone	167(90.8)	164(88.2)	159(86.4)	0.423
Dexamethasone	1(0.5)	5(2.7)	2(1.1)	0.291
Intercostal nerve block, no. (%)	149(81.0)	148(79.6)	139(75.5)	0.417
Total fluid, mL/kg/h, median (IQR)	2.9(2.2,3.9)	3.1(2.3,3.9)	3.1(2.2,3.9)	0.777
Pain score in PACU, median (IQR)	1(0,2)	1(0,2)	1(0,2)	0.752
Rescue analgesics in PACU, no. (%)	5(2.7)	5(2.7)	7(3.8)	0.778
PONV in PACU, no. (%)				0.850
0	183(99.5)	185(99.5)	183(99.5)	
1	1(0.5)	0	1(0.5)	
2	0	0	0	
3	0	1(0.5)	0	
4	0	0	0	

Data are presented as the median (IQR) or number (%)

Abbreviations: *IQR* interquartile range, *NSAIDS* non-steroidal anti-inflammatory drugs, *PACU* Post-anesthesia care unit, *PONV* postoperative nausea and vomiting

### Primary outcome

The primary outcome of SAME was significantly different among groups O, H and S (41.3% vs 40.3% vs 29.9%, *P* = 0.043), but no difference was observed between pairwise group comparisons; The SAME on POD 1 were significantly different among three groups (42.4% *vs* 40.9% *vs* 30.4%, *P* = 0.037), but no difference was observed between pairwise group comparisons; On POD 2, groups O (59.2% *vs* 43.5%, *P* = 0.007) and H (57.2% vs 43.5%, *P* = 0.02) had significantly more patients achieving SAME than group S; On POD 3, the proportion of patients achieving SAME were similar among three groups (90.2% *vs* 86.6% *vs* 86.4%, *P* = 0.452) (Table 3). For per-protocol analysis, the primary outcome had no change (Additional file 1).

**Table 3:** Comparison of postoperative outcomes in the intention-to-treat analysis

Outcomes	Oxycodone group (*n* = 184)	Hydromorphone group (*n* = 186)	Sufentanil group (*n* = 184)	*P* value	*P* value O versus H/ O versus S/ H versus S
**Primary outcome, SAME on cough, no. (%)**	76(41.3)	75(40.3)	55(29.9)	0.043	1.000/0.071/0.114
POD 1–3 Pain score < 4 on cough, no. (%)	79(42.9)	77(41.4)	56(30.4)	0.027	1.000/0.041/0.091
POD 1–3 PONV score < 2, no. (%)	173(94.0)	168(90.3)	167(90.8)	0.372	n/a
POD 1 SAME on cough, no. (%)	78(42.4)	76(40.9)	56(30.4)	0.036	1.000/0.055/0.117
POD 2 SAME on cough, no. (%)	109(59.2)	107(57.5)	80(43.5)	0.004	1.000/0.007/0.020
POD 3 SAME on cough, no. (%)	166(90.2)	161(86.6)	159(86.4)	0.451	n/a
**Secondary outcomes**					
SAME at rest, no. (%)	168(91.3)	162(87.1)	158(85.9)	0.240	n/a
POD 1–3 Pain score < 4 at rest, no. (%)	177(96.2)	175(94.1)	173(94.0)	0.566	n/a
POD 1 SAME at rest, no. (%)	168(91.3)	162(87.1)	158(85.9)	0.240	n/a
POD 2 SAME at rest, no. (%)	182(98.9)	183(98.4)	181(98.4)	1.000	n/a
POD 3 SAME at rest, no. (%)	183(99.5)	186(100.0)	184(100.0)	0.664	n/a
Total dose of opioid in morphine equivalents, mg, median (IQR)	29.5(14.0,48.0)	25.0(12.8,45.3)	36.5(18.0,55.8)	0.006	0.734/0.134/0.004
Dose of opioid of PCA in morphine equivalents, mg, median (IQR)	20.0(10.0,37.5)	20.0(10.0,28.0)	24.0(12.0,42.0)	0.009	0.416/0.342/0.007
Rescue analgesics during POD 1–3, no. (%)	10(5.4)	11(5.9)	16(8.7)	0.400	n/a
Patient satisfaction score on pain control, median (IQR)	97(93,100)	96(92,100)	97(92,100)	0.768	n/a
QoR-15 score, median (IQR)					
POD 1	126(121,130)	126(120,130)	126(119,130)	0.561	n/a
POD 2	133(130,136)	132(129,135)	132(129,135)	0.217	n/a
POD 3	139(136,140)	138(136,140)	138(136,140)	0.718	n/a
Chest tube duration, days, median (IQR)	2(2,3)	2(2,4)	2(2,3)	0.094	n/a
Discharge time from hospital, days, median (IQR)	3(3,4)	4(3,5)	3.5(3,5)	0.041	0.043/0.227/1.000
Other opioid-related adverse events, no. (%)					
Constipation	66(35.9)	57(30.6)	65(35.3)	0.506	n/a
Dizziness	50(27.2)	45(24.2)	32(17.4)	0.073	n/a
Pruritis	2(1.1)	3(1.6)	1(0.5)	0.875	n/a
Urinary retention	6(3.3)	9(4.8)	10(5.4)	0.583	n/a
Severe sedation^a^	0	0	0	n/a	n/a
Respiratory depression	0	0	0	n/a	n/a
PCA withdrawal due to adverse events	6(3.3)	11(5.9)	7(3.8)	0.416	n/a

Data are presented as the median (IQR) or number (%)

Abbreviations: *IQR* interquartile range, *PCA* patient-controlled analgesia, *POD* postoperative day, *PONV* postoperative nausea and vomiting, *QoR-15* Quality of Recovery-15 questionnaire, *SAME* satisfactory analgesia with minimal emesis.

^a^defined as Ramsay sedation scale score of 5–6

### Key secondary outcomes

**Fig. 2:**
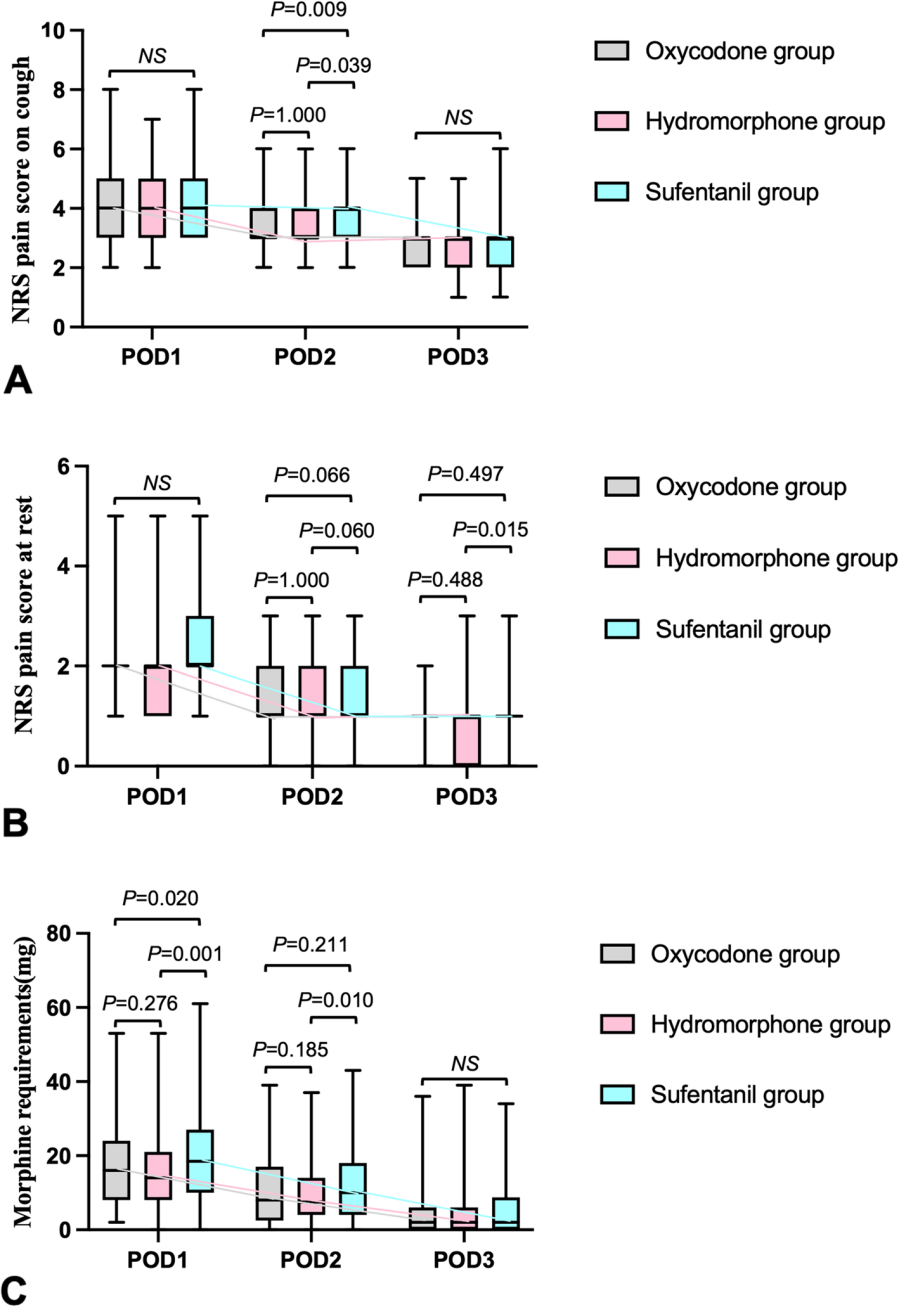
Comparison of NRS pain scores and morphine requirements within 3 days after surgery among three groups. NRS, numerical rating scale; POD, postoperative day. Top and bottom of boxes indicate interquartile range; centerlines indicate medians; whiskers indicate range (minimum to maximum)

**Fig. 3:**
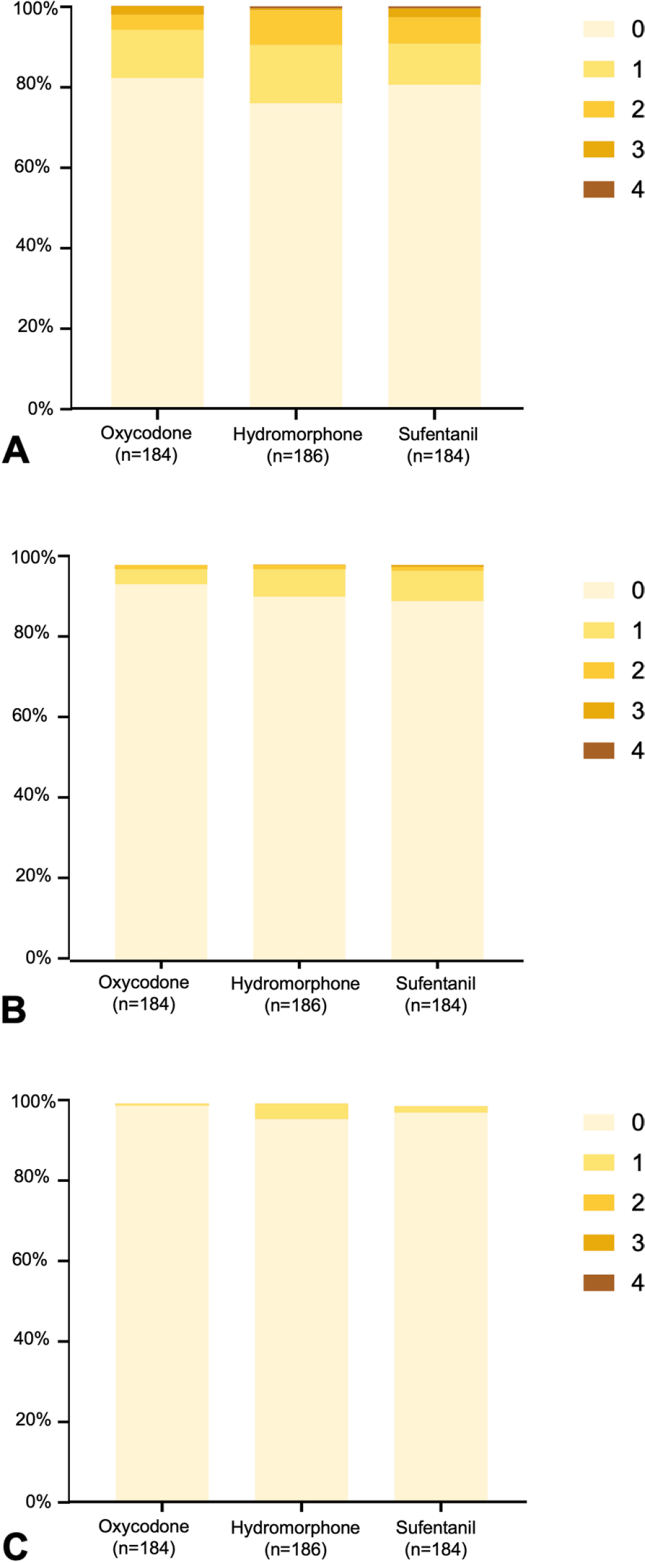
Comparison of PONV score among three groups. POD, postoperative day; PONV, postoperative nausea and vomiting. **A** POD 1; **B** POD 2; **C** POD 3

Patients in groups O and H had lower pain scores when coughing on POD2 than those in group S, both with mean differences of 1 (3(3,4) and 3(3,4) *vs* 4(3,4), *P* = 0.009 and 0.039, respectively) (Fig. 2). The PONV scores within 3 days after surgery were comparable among three groups (*P* > 0.05) (Fig. 3).

### Other secondary outcomes

The SAME ratio at rest was similar during POD 1–3 (91.3% in group O *vs* 87.1% in group H *vs* 85.9% in group S, *P* = 0.24). Group H took less opioid both in total dose (25.0(12.8,45.3) mg *vs* 36.5(18.0,55.8) mg, *P* = 0.004) and in PCIA (20.0(10.0,28.0) mg *vs* 24.0(12.0,42.0) mg, *P* = 0.007) in morphine equivalents than group S. Subjects in all groups achieved a very good satisfaction score and QoR-15 score. Other opioid-related adverse events also were not significantly different among groups. Group O had shorter LOS after surgery than Group H (3(3,4) days vs 4(3,5) days, *P* = 0.043). The expectation NRS fulfilled (on cough, at rest, on average) within 3 days after surgery were comparable among three groups (*P* > 0.05) (Additional file 2).

## Discussion

In this randomized clinical study, we found that the proportion of subjects achieving SAME within the first 3 days was of statistical significance among oxycodone, hydromorphone and sufentanil for PCIA in patients undergoing thoracoscopic lung surgery. On POD 2, oxycodone and hydromorphone had significantly more patients achieving SAME than sufentanil; the pain scores when coughing on POD 2 were significantly lower in patients receiving hydromorphone or oxycodone than sufentanil; the PONV scores and other opioid-related adverse events within 3 days after surgery were comparable among three groups.

Perioperative pain management becomes increasingly important to the quality of surgical care [3]. Postoperative pain reduction is therefore one of the key elements of enhanced recovery after surgery (ERAS) program. Epidural analgesia may provide excellent pain relief, but is considered to be less necessary for a variety of less invasive surgical procedures, such as VATS [21]. Multimodal analgesia techniques, such as regional analgesia [22, 23], local anesthesia [24] and systemic analgesia are recommended [9]. Systemic opioids for pain treatment still gets mentioned in the latest WHO list of essential medicines for perioperative analgesia [10, 25]. Additionally, the opioid epidemic that may have originated in the US and Europe is not significant in China [10]. Considering a technical simplicity and less invasiveness of PCIA, this analgesic approach may be worthy of further research and is thus considered to have potential as a viable alternative to postoperative analgesia. So far, the existing literature is unclear about the choice of systemic opioid analgesia to be used postoperatively in thoracoscopic lung surgery [9].

To our knowledge, our trial is the first RCT to provide new evidence of PCIA with opioids (oxycodone, hydromorphone and sufentanil). Considering the analgesic efficacy and the adverse event profile of opioids, our study focused on the proportion of satisfactory analgesia with minimal emesis within the first 3 days after surgery. We found a 38% relative (11.4% absolute), or 35% relative (10.4% absolute) increase in the proportion of patients achieving SAME when compared oxycodone or hydromorphone with sufentanil, respectively, which was not statistically significant but, in our view, clinically relevant. Of note, more than half of patients receiving oxycodone (59.2%) or hydromorphone (57.5%) achieving SAME in the second day after surgery, and the differences reached statistically significant when compared to patients receiving sufentanil (43.5%). In addition, oxycodone or hydromorphone was superior to sufentanil for acute pain when comparing pain scores when coughing. In our view, this finding supported that the oxycodone or hydromorphone was superior to sufentanil for PCIA because clinically relevant benefits were detected. However, the existing studies that have compared oxycodone, hydromorphone and sufentanil in surgical patients are limited. A recent meta-analysis evaluated the acute postoperative analgesic efficacy of intravenous oxycodone against other strong opioids in adult patients [26]. There were only three studies comparing oxycodone with sufentanil, and no eligible studies comparing oxycodone with hydromorphone. Consistent with our finding, they observed oxycodone exhibited better analgesic efficacy than sufentanil, with comparable incidence of nausea.

Although PONV is typically considered an inherent side-effect of opioid-based analgesia, our study showed that more than 90% patients had no or mild nausea (PONV score < 2) within the first 3 days after surgery. A previous study reported that a lower dose background infusion of oxycodone was associated with fewer PONV [27]. The relatively lower incidence of PONV found in our trial may be explained by no background infusion dose of opioids compared with published literature [28, 29]. Additionally, we found no significant difference among three study groups regarding PONV. This finding was consistent with a meta-analysis which showed that oxycodone, hydromorphone or sufentanil in equianalgesic doses via PCIA had no significant difference regarding PONV rates compared to morphine [15].

Our findings suggested that the incidence of other opioid-related adverse events was not significantly different among oxycodone, hydromorphone and sufentanil. Our reported incidence of constipation (33.9%) and dizziness (22.9%) were similar to Lee et al.’s study, in which constipation (28.9%) and dizziness (21.1%) were two most common side effects with opioid PCIA after thoracoscopic lobectomy [29]. Pruritus (1.1%) or urinary retention (4.5%) was observed in a small proportion of the study population, which was relatively lower compared to other literatures using fentanyl [29], morphine [30] or hydromorphone [31]. No sedation or respiratory depression was observed in our trial, which was more common with morphine [32, 33]. Although the adverse events occurred in a various proportion of patients, subjects in all groups achieved a very good satisfaction score, QoR-15 score and expectation NRS fulfilled rate. Given that, our study supports that PCIA with systemic opioids is the effective and well tolerated approach with minimum adverse effects and good patient satisfaction.

This study had several limitations. First, as the treatment effect of oxycodone or hydromorphone was lower than anticipated (a relative increase of 50%) compared to sufentanil, the trial was possibly underpowered to assess the primary outcome, and therefore, should be considered a pilot study. Therefore, no strong conclusions could be drawn from the present work. Consequently, further studies are needed to confirm the superiority of oxycodone and hydromorphone when compared to sufentanil for PCIA. Second, a small proportion of patients were not strictly following the optimal pain treatments recommended by PROSPECT for VATS, including intercostal nerves block (21%) and NSAIDs intra-operatively (8.8%) [9]. However, the use of these treatments was similar among three groups. Additionally, in our trial, the average pain scores within the first 3 days were equal or less than 4 (considered the upper end of mild pain category), and about only 5% of patients suffered moderate-to-severe pain at rest, so the basic analgesic protocol in this trial could be optimal.

## Conclusions

Given clinically relevant benefits detected, PCIA with oxycodone or hydromorphone was superior to sufentanil for achieving satisfactory analgesia with minimal emesis in patients undergoing thoracoscopic lung surgery. The PCIA with opioids is considered to have potential as a supplement to multimodal analgesia with technical simplicity and less invasiveness, and may be worthy of further research.

## Supplementary Information

**Additional file 1.** Comparison of postoperative outcomes in the per-protocol analysis. Dataare presented as the median (IQR) or number (%). Abbreviations: IQR, interquartilerange; PCIA, patient-controlled intravenous analgesia; POD, postoperative day;PONV, postoperative nausea and vomiting; QoR-15, Quality of Recovery-15questionnaire; SAME, satisfactory analgesia with minimal emesis.

**Additional file 2.** Expectation on pain fulfilled rate. POD, postoperativeday. A. Expectation on cough fulfilled; B. Expectation at rest fulfilled; C.Expectation on average pain fulfilled.

## Data Availability

The datasets generated and analyzed during the current study are available from the corresponding author on reasonable request.

## Abbreviations

ASA: American Society of Anesthesiologists
BIS: Bispectral index
BMI: Body mass index
CONSORT: Consolidated Standards of Reporting Trials
EtCO_2_: End-expiratory CO_2_
FiO_2_: Fraction inspired oxygen
I:E: Inspiratory-expiratory ratio
IQR: Interquartile range
ITT: Intent-to-treat
LOS: Length of stay
NRS: Numerical rating scale
NSAIDS: Non-steroidal anti-inflammatory drugs
OLV: One-lung ventilation
PACU: Post-anesthesia care unit
PCIA: Patient-controlled intravenous analgesia
PEEP: Positive end-expiratory pressure
POD: Postoperative day
PONV: Postoperative nausea and vomiting
PP: Per-protocol
QoR-15: Quality of Recovery-15 questionnaire
RCT: Randomized controlled trial
SAME: Satisfactory analgesia with minimal emesis
SD: Standard deviation
VATS: Video-assisted thoracoscopic surgery
VT: Tidal volume
