# Supplementary material for: Patient-controlled intravenous analgesia with opioids after thoracoscopic lung surgery: a randomized clinical trial
Source: BMC Anesthesiol. 2022 Aug 8;22:253. doi: 10.1186/s12871-022-01785-4 (PMC9358799; doi:10.1186/s12871-022-01785-4)
Supplement: Supplementary file 1 — Additional file 1. Comparison of postoperative outcomes in the per-protocol analysis. Dataare presented as the median (IQR) or number (%). Abbreviations: IQR, interquartilerange; PCIA, patient-controlled intravenous analgesia; POD, postoperative day;PONV, postoperative nausea and vomiting; QoR-15, Quality of Recovery-15questionnaire; SAME, satisfactory analgesia with minimal emesis. [file 12871_2022_1785_MOESM1_ESM.docx]

| **Supplemental Table S1 Comparison of postoperative outcomes in the per-protocol analysis** | | | | | |
| --- | --- | --- | --- | --- | --- |
| **Outcomes** | **Oxycodone group（n=183）** | **Hydromorphone group（n=186）** | **Sufentanil group（n=183）** | **P value** | **P value**  **O versus H/ O versus S/ H versus S** |
| **Primary outcome** |  |  |  |  |  |
| POD1-3 SAME on cough, no. (%) | 76(41.5) | 75(40.3) | 55(30.1) | 0.044 | 1.000/0.070/0.125 |
| POD1 | 78(42.6) | 76(40.9) | 56(30.6) | 0.038 | 1.000/0.054/0.128 |
| POD2 | 108(59.0) | 107(57.5) | 80(43.7) | 0.005 | 1.000/0.010/0.024 |
| POD3 | 165(90.2) | 161(86.6) | 158(86.3) | 0.457 | n/a |
| POD 1-3 Pain score＜4 on cough, no. (%) | 79(43.2) | 77(41.4) | 56(30.6) | 0.028 | 1.000/0.041/0.099 |
| POD 1-3 PONV score＜2, no. (%) | 172(94.0) | 168(90.3) | 166(90.7) | 0.377 | n/a |
| **Secondary outcomes** |  |  |  |  |  |
| POD1-3 SAME at rest, no. (%) | 167(91.3) | 162(87.1) | 157(85.8) | 0.242 | n/a |
| POD1 | 167(91.3) | 162(87.1) | 157(85.8) | 0.242 | n/a |
| POD2 | 181(98.9) | 183(98.4) | 180(98.4) | 1.000 | n/a |
| POD3 | 182(99.5) | 186(100) | 183(100) | 0.663 | n/a |
| POD 1-3 Pain score＜4 at rest, no. (%) | 176(96.2) | 175(94.1) | 172(94.0) | 0.570 | n/a |
| Total dose of opioid in morphine equivalents, mg, median (IQR) | 29.0(14.0,48.0) | 25.0(12.8,45.3) | 36.0(18.0,55.0) | 0.007 | 0.843/0.131/0.006 |
| Dose of opioid of PCIA in morphine equivalents, mg, median (IQR) | 20.0(10.0,36.0) | 20.0(10.0,28.0) | 24.0(12.0,42.0) | 0.012 | 0.467/0.360/0.009 |
| Rescue analgesics during POD 1-3, no. (%) | 10(5.5) | 11(5.9) | 16(8.7) | 0.396 | n/a |
| Patient satisfaction score on pain control, median (IQR) | 96(93,100) | 96(92,100) | 97(92,100) | 0.784 | n/a |
| QoR-15 score, median (IQR) |  |  |  |  |  |
| POD1 | 126(121,130) | 126(120,130) | 126(119,130) | 0.589 | n/a |
| POD2 | 133(130,136) | 132(129,135) | 132(129,135) | 0.231 | n/a |
| POD3 | 139(136,140) | 138(136,140) | 138(136,140) | 0.740 | n/a |
| Chest tube duration, days, median (IQR) | 2(2,3) | 2(2,4) | 2(2,3) | 0.082 | n/a |
| Discharge time from hospital, days, median (IQR) | 3(3,4) | 4(3,5) | 4(3,5) | 0.033 | 0.036/0.180/1.000 |
| Other opioid-related adverse events, no. (%) |  |  |  |  |  |
| Constipation | 65(35.5) | 57(30.6) | 65(35.5) | 0.520 | n/a |
| Dizziness | 50(27.3) | 45(24.2) | 32(17.5) | 0.074 | n/a |
| Pruritis | 2(1.1) | 3(1.6) | 1(0.5) | 0.875 | n/a |
| Urinary retention | 6(3.3) | 9(4.8) | 10(5.5) | 0.585 | n/a |
| Severe sedation^a^ | 0 | 0 | 0 | n/a | n/a |
| Respiratory depression | 0 | 0 | 0 | n/a | n/a |
| PCIA withdrawal due to adverse events | 6(3.3) | 11(5.9) | 7(3.8) | 0.423 | n/a |
| Data are presented as the median (IQR) or number (%).  Abbreviations: IQR, interquartile range; PCIA, patient-controlled intravenous analgesia; POD, postoperative day; PONV, postoperative nausea and vomiting; QoR-15, Quality of Recovery-15 questionnaire; SAME, satisfactory analgesia with minimal emesis.  a defined as Ramsay sedation scale score of 5-6. | | | | | |
