# Supplementary figures and images for: Patient-controlled intravenous analgesia with opioids after thoracoscopic lung surgery: a randomized clinical trial
Source: BMC Anesthesiol. 2022 Aug 8;22:253. doi: 10.1186/s12871-022-01785-4 (PMC9358799; doi:10.1186/s12871-022-01785-4)

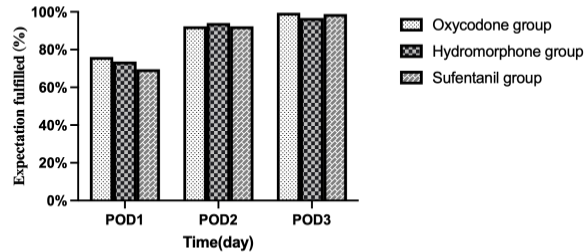

**A**

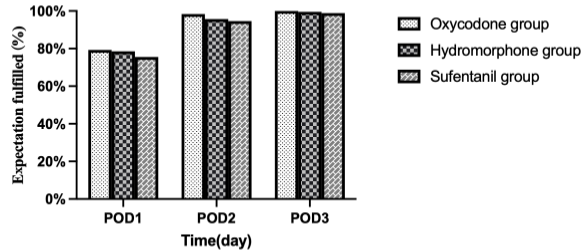

**B**

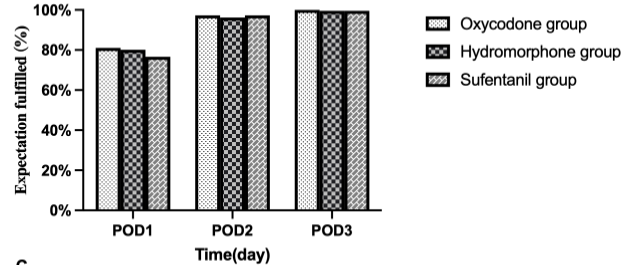

**C**

**Supplemental Figure S1** Expectation on pain fulfilled rate. POD, postoperative day.

Supplement: Supplementary file 2 — Additional file 2. Expectation on pain fulfilled rate. POD, postoperativeday. A. Expectation on cough fulfilled; B. Expectation at rest fulfilled; C.Expectation on average pain fulfilled. [file 12871_2022_1785_MOESM2_ESM.pdf]
